# Improving Breast Cancer Treatment Specificity Using Aptamers Obtained by 3D Cell-SELEX

**DOI:** 10.3390/ph14040349

**Published:** 2021-04-09

**Authors:** Frank H. T. Nelissen, Wenny J. M. Peeters, Timo P. Roelofs, Anika Nagelkerke, Paul N. Span, Hans A. Heus

**Affiliations:** 1 Institute for Molecules and Materials, Radboud University, 6525 AJ Nijmegen, The Netherlands; Frank.Nelissen@ru.nl (F.H.T.N.); T.Roelofs@student.ru.nl (T.P.R.); 2 Radiotherapy & OncoImmunology Laboratory, Department of Radiation Oncology, Radboud Institute for Molecular Life Sciences, Radboud University Medical Center, 6525 GA Nijmegen, The Netherlands; Wenny.Peeters@radboudumc.nl; 3 Faculty of Science and Engineering, Pharmaceutical Analysis—Groningen Research Institute of Pharmacy, 9713 AV Groningen, The Netherlands; a.p.nagelkerke@rug.nl

**Keywords:** aptamer, breast cancer, SKBR3, spheroids, doxorubicin, multivalency

## Abstract

Three-dimensional spheroids of non-malignant MCF10A and malignant SKBR3 breast cells were used for subsequent 3D Cell-SELEX to generate aptamers for specific binding and treatment of breast cancer cells. Using 3D Cell-SELEX combined with Next-Generation Sequencing and bioinformatics, ten abundant aptamer families with specific structures were identified that selectively bind to SKBR3, and not to MCF10A cells. Multivalent aptamer polymers were synthesized by co-polymerization and analyzed for binding performance as well as therapeutic efficacy. Binding performance was determined by confocal fluorescence imaging and revealed specific binding and efficient internalization of aptamer polymers into SKBR3 spheroids. For therapeutic purposes, DNA sequences that intercalate the cytotoxic drug doxorubicin were co-polymerized into the aptamer polymers. Viability tests show that the drug-loaded polymers are specific and effective in killing SKBR3 breast cancer cells. Thus, the 3D-selected aptamers enhanced the specificity of doxorubicin against malignant over non-malignant breast cells. The innovative modular DNA aptamer platform based on 3D Cell SELEX and polymer multivalency holds great promise for diagnostics and treatment of breast cancer.

## 1. Introduction

Breast cancer is the most frequently diagnosed type of cancer among women, as well as the leading cause of cancer death. World-wide, over two million new cases of breast cancer were estimated in 2018, accounting for nearly 25% of all cancer cases, and over 600,000 deaths were reported due to breast cancer [1]. Breast cancer is a highly heterogeneous disease, and several classifications for identifying subtypes of breast cancer have been developed, based on histological and molecular markers [2]. Currently, several subtypes are classified for medical practice, which are mainly distinguished on the basis of the expression of three key receptors: The hormone receptors estrogen (ER) and progesterone (PR), and the human epidermal growth factor 2 (HER2). Adjuvant treatment is based on these subtypes, and may consist of anti-hormonal, anti-HER2, and/or chemotherapy [2]. Chemotherapy, often consisting of doxorubicin, sometimes combined with cyclophosphamide and/or 5-fluorouracil, is, however, limited by toxicity [3] and lacks specificity. Therefore, continued research is necessary for further development of drugs, designed to target specific breast cancer cells as well as for developing novel tools for monitoring patients and early detection of breast cancer. 

Aptamers are single-stranded DNA or RNA oligonucleotides that can be generated using a selection process called SELEX (Systematic Evolution of Ligands by EXponential enrichment) to specifically bind with high affinity to molecular targets [4,5]. Aptamers can be selected for virtually every possible target, including metal ions, small molecules such as drugs or peptides, proteins, viruses, bacteria, whole cells, or even targets within living animals (reviewed in [6,7,8,9]). The affinity and specificity of aptamers is comparable to traditional antibodies, and aptamers are a proven alternative to antibodies for activating or inhibiting processes, or to serve as vehicles for targeted delivery of imaging or therapeutics agents. Moreover, compared to antibodies, aptamers have several advantages, such as ease of synthesis, modification and functionalization, high stability, and low toxicity and immunogenicity. This makes aptamers ideal tools for the development of novel strategies in selective diagnostics and therapeutics of cancer. 

Aptamers have been generated using traditional SELEX technology against purified proteins or whole cells to select for targets exposed on cell surfaces. In this study, we used 3D spheroids as targets for the SELEX procedure to generate aptamers against tumor cells in a more physiologically relevant microenvironment. We identified DNA aptamers that bind with high affinity and specificity to SKBR3 spheroid breast cancer cells. Using a modular polymeric approach, we constructed multivalent aptamer polymers with DNA boxes carrying doxorubicin payloads that are effective and specific in killing target breast cancer cells. The 3D spheroid cell SELEX and modular synthesis approaches hold great promise as novel strategies for developing tools for cancer diagnostics and therapeutics. 

## 2. Results

### 2.1. Selection of Aptamers against SKBR3 Spheroids 

**Figure 1:**
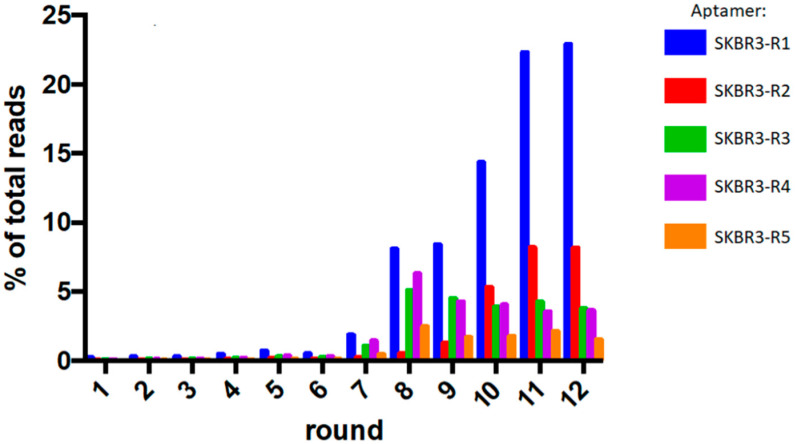
Graphical representation of the enrichment of the top 5 raised aptamer sequences in each round of the selection procedure.

A novel 3D Cell-SELEX procedure was used to generate DNA aptamers, using tight and compact spheroids of SKBR3 breast cancer cells as target [10]. Multicellular tumor spheroid models have been widely used to mimic the three-dimensionality of solid tumors [11]. However, with one exception, i.e., for selection of aptamers against prostate cancer cells [12], spheroids have so far not been used as targets for selection of aptamers. Compact, tight spheroids of SKBR3 breast cancer cells were used as targets and non-malignant MCF10A breast epithelial cells for negative selection during the SELEX procedure. Spheroids were grown in U-shaped 96-well plates using the liquid overlay technique with Matrigel addition [10]. For the selection process, we used a 91 nucleotide DNA oligonucleotide containing two 23-nucleotide random sequences (Supplementary Table S1). The two random regions were separated by a stable GAA hairpin loop to force the aptamers into a more stable fold for enhanced binding. In the first two rounds of the selection procedure, the aptamer pool was targeted to monolayers of SKBR3 cells and control MCF10A cells, grown to confluency. From the third round on, spheroids were used for selection. A total of 12 rounds of SELEX were performed (Supplementary Table S2), after which sequences were analyzed using Next Generation Sequencing (NGS) methods. First, the top 100 most frequent sequences were grouped in families based on sequence homology with the top 10 most frequent sequences using the program ClustalX (Table 1, Supplementary Table S3) [13]. Secondary structures of the 10 most frequent sequences of the final round were analyzed using M-Fold [14]. This analysis shows formation of stable double-stranded regions connected by internal and hairpin loops, as well as large unstructured regions (Supplementary Figure S1). The sequences can be grouped in 10 different families based on distinct secondary structure features. Samples of all rounds were sequenced to monitor the selection process. As can be seen in Figure 1, the sequence space converges after round 6, finally resulting in 5 dominant species, which are already present at 0.04 – 0.2% in the first few rounds. In the final round the top 5 most frequent sequences account for 40% of all sequences (Supplementary Table S4).

**Figure 2:**
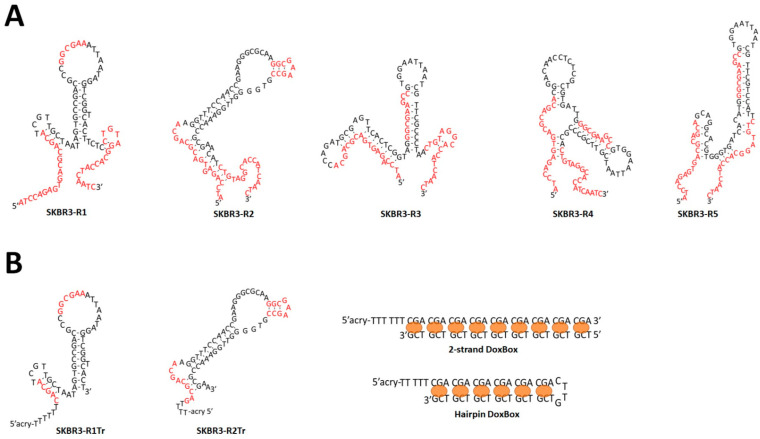
*(***A**) Predicted secondary structures of the top 5 raised aptamers. Structures were obtained with the DNA folding module on the Mfold webserver [14] using ionic strength conditions of 150 mM NaCl and 5 mM Mg^2+^ at 20 °C. Nucleotides in red originate from library design. (**B**) Predicted secondary structures of truncated SKBR3-R1 and SKBR3-R2 aptamers and hairpin Dox box and 2-strand Dox box sequences for acrylamide copolymerization. Doxorubicin binding sites are indicated with orange ovals.

The stable secondary structures of the 5 dominant sequences, of which each structure represents one distinct structural family, are shown in Figure 2. Interestingly, only one sequence, SKBR3-R2, is predicted to form the input stable GAA hairpin loop, and the hairpin sequence is partially deleted in SKBR3-R1. This illustrates the power of selection from a library of random sequences, which apparently overrules rational design. 

For in vivo applications and labeling strategies for imaging and stability purposes, the availability of short sequences, without compromising affinity and specificity, is an important issue. Further secondary structure analysis revealed common structural signatures among the different families. Assuming that affinity and specificity of aptamers depend on their specific shapes [5,15,16], these common structural signatures can be used to design truncated versions of the selected aptamers. The truncated versions, obtained by omitting the flanking constant sequences used for amplification and sequencing, are also shown in Figure 2. SKBR3-R1Tr, which is a derivative of SKBR3-R1, was designed because it represents the most abundant selected sequence (22.9% in the final, 12th round). SKBR3-R2Tr was designed because of its atypical loop motif. These truncated versions were used for most further studies.

### 2.2. Affinity and Specificity of Aptamers

**Figure 3:**
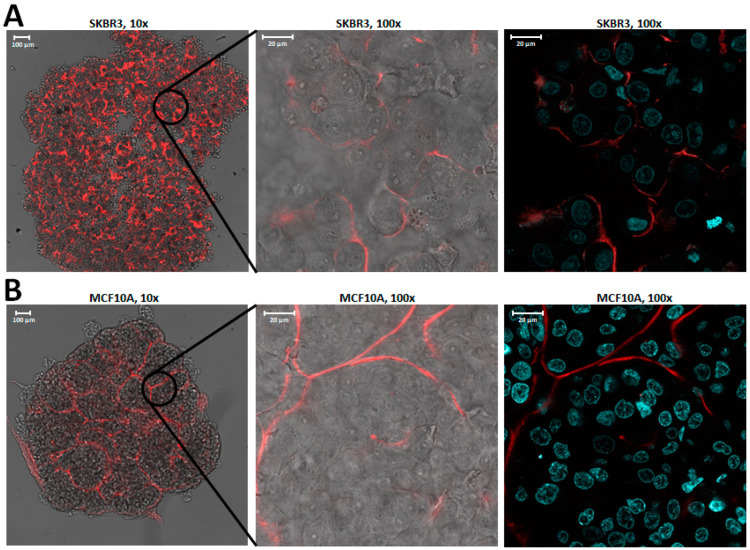
Images of spheroids incubated with full SKBR3-R1 aptamers labeled with Alexa Fluor 594 after one hour incubation at room temperature. Images on the left are at 10×, images in the middle at 10× magnification, bright field, and AF594 signal (red). The images on the right show Hoechst-stained nuclei (cyan) and the AF594 signal (red). Panel (**A**), SKBR3 spheroids. Panel (**B**), MCF10A spheroids.

To assess the affinity and specificity of the selected aptamers for cancer cells, DNAs were synthesized with a 3’ribose for labeling with fluorescent dyes (Alexa Fluor 488 or 594) using NaIO_4_–hydrazide chemistry. Specificity of aptamer binding to 3D spheroids of target SKBR3 cells and control MCF10A cells was initially assessed using fluorescence microscopy (Figure 3). The full SKBR3-R1 aptamers efficiently bind to the target SKBR3 breast cancer cells, as shown by formation of tight coronas of bound fluorescent aptamers around the target cells after 1 h incubation at room temperature (Figure 3A, Supplementary Figure S2). The control MCF10A do not show formation of coronas of fluorescence around the cells, indicating absence of binding. Here, the fluorescent aptamers do not bind to the cells in the spheroids, but only freely diffuse in the channels formed in between the spheroid cells (Figure 3B). A randomized aptamer (Scramble-AF594) that was used as control, did not show binding to either SKBR3 or MCF10A spheroids (Supplementary Figure S3). To further illustrate the affinity of the selected aptamers for target SKBR3 cells, the binding affinity of SKBR3-R1Tr to SKBR3 spheroids was determined by serial dilution of the aptamer. The data reveal high affinity with a Kd of 81.4 nM (Supplementary Figure S4). 

### 2.3. Internalization of Aptamers

**Figure 4:**
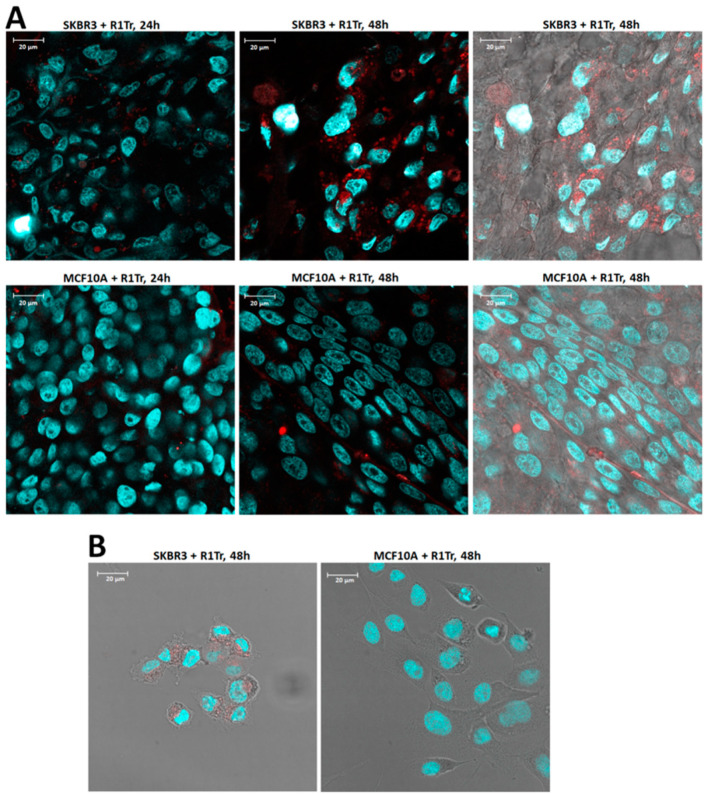
Panel (**A**) Images of SKBR3 spheroids (top row) and MCF10A spheroids (bottom row) incubated with truncated SKBR3-R1Tr-AF594 aptamers. Spheroids in the images on the left were incubated for 24 h with aptamers and spheroids in the middle images for 48 h. The images on the right include the bright field images of the 48 h incubations. Panel (**B**) Images of SKBR3 cells (left) and MCF10A cells (right) grown in 2D in chamber slides, incubated with truncated SKBR3-R1Tr-AF594 aptamers. Cells were incubated for 48 h with aptamers and images were acquired at 100× magnification.

The observed specificity of binding shows the potential of the aptamers as an important tool for diagnostics of breast cancer involving SKBR3 cells, a cell line representative of HER2+ breast cancer. However, for therapeutic purposes such as drug delivery or siRNA delivery using aptamers as a carrier, it is important to show that the selected aptamers are internalized into the target cells after binding [17]. Therefore, further fluorescence microscopy experiments were performed to investigate if the aptamers internalize into target cells. At 37 °C, the SKBR3-R1Tr aptamers readily internalize into SKBR3 spheroid cells, as shown by the typical punctate pattern of a large number of clear fluorescent spots in the microscope images after 24–48 h of incubation (Figure 4A). The aptamers do not internalize into control MCF10A cells, but also here only freely diffuse in the channels formed inside the spheroid. The control, randomized aptamer Scramble-AF594 did not internalize into SKBR3 spheroids (Supplementary Figure S5). Specificity of binding is further illustrated by internalization of SKBR3-R1Tr aptamers into SKBR3 cells grown as 2D monolayers and complete absence of internalization into control MCF10A cells grown in 2D (Figure 4B). Importantly, the intensity of aptamers bound to SKBR3 cells in 2D tissue cultures is apparently considerably lower than 3D spheroids, indicating higher affinity for cells in 3D spheroids. To investigate the mechanism of internalization, experiments were conducted using fluorescent-labelled dextran, which is a marker of macropinocytosis [18,19]. Fluorescent-labelled dextran is taken up by the target SKBR3 cells as well as control MCF10A cells (Supplementary Figure S6). Fluorescent-labelled aptamers mixed with fluorescent-labelled dextran are only taken up by SKBR3 and co-localize with the dextran label, indicating a macropinocytosis type of mechanism for internalization.

### 2.4. Drug Delivery Using Polymeric Payloads

**Figure 5:**
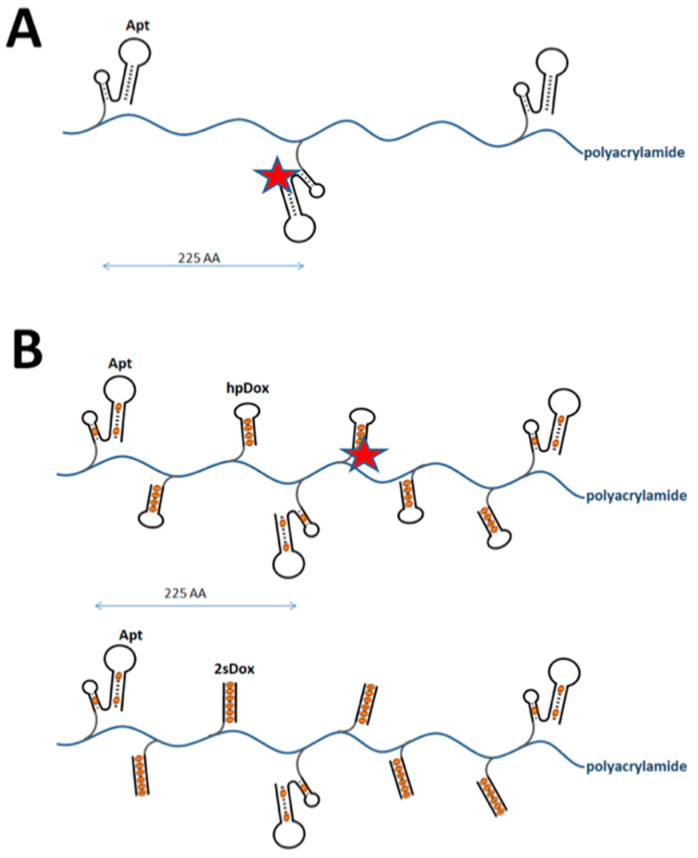
(**A**) Schematic of truncated SKBR3-R1 (Apt) co-polymerized with acrylamide. Spacing between each aptamer is ~225 acrylamide monomers and one in every five aptamers is labeled with Alexa Fluor 594 (red star). (**B**) Schematic of truncated SKBR3-R1 (Apt) co-polymerized with hairpin Dox Boxes (hpDox) or 2-strand Dox boxes (2sDox) and acrylamide in a ratio 1:2.5:225. In the case of the hairpin Dox boxes, one in every 10 Dox boxes is labeled with Alexa Fluor 594 (red star). Dox is indicated as orange ovals.

Doxorubicin (Dox) is one of the most potent chemotherapeutic drugs developed, and has been used in a variety of anticancer treatment including breast cancer [20]. Dox acts by intercalating DNA, which causes disruption of DNA replication and finally results in apoptotic cell death. Unfortunately, the treatment of breast cancer using systemic administration of Dox is severely hampered by limited maximal dosage, acquired drug resistance, and serious side effects, in particular cardiotoxicity [3]. The mechanism of Dox toxicity, however, which involves intercalation of specific DNA sequences, can be harnessed for targeted delivery of Dox using DNA aptamer technology. Aptamers can be extended with short hairpins or double stranded DNA regions containing CpG stretches, which are known to efficiently intercalate Dox [21,22,23,24]. Instead of extending aptamers with DNA Dox carriers for targeted delivery, which has been the approach by various laboratories [25,26,27,28,29,30,31,32,33,34,35], here we use a modular approach, where we combine Dox carriers with high payload capacity with our selected aptamers using polymer synthesis, as schematized in Figure 5. 

Polyacrylamide was used as the polymer backbone based on its stability and biocompatibility [36,37]. Acrydite-modified oligonucleotides were used to incorporate aptamers and Dox carriers (Dox boxes) into polyacrylamide during polymerization. Three different polymers were synthesized. Multivalent aptamer polymers were synthesized with either SKBR3-R1Tr1 or SKBR3-R1Tr2 using a ratio of 1:225 (aptamer:acrylamide monomer, Figure 5A). Dox boxes were incorporated as double-stranded (2sDox) or hairpin (hpDox) modules using a ratio of 1:2.5:225 (DNA:Dox:acrylamide). For microscopic imaging experiments, Alexa Fluor 594 (AF594) modified aptamers (ratio 1:5) or fluorophore modified hairpin Dox boxes (ratio 1:10) were co-polymerized. The sequences of the Dox boxes were designed such that each hairpin module contains six intercalation sites for Dox loading, and each double-stranded Dox box nine (Figure 2B). 

**Figure 6:**
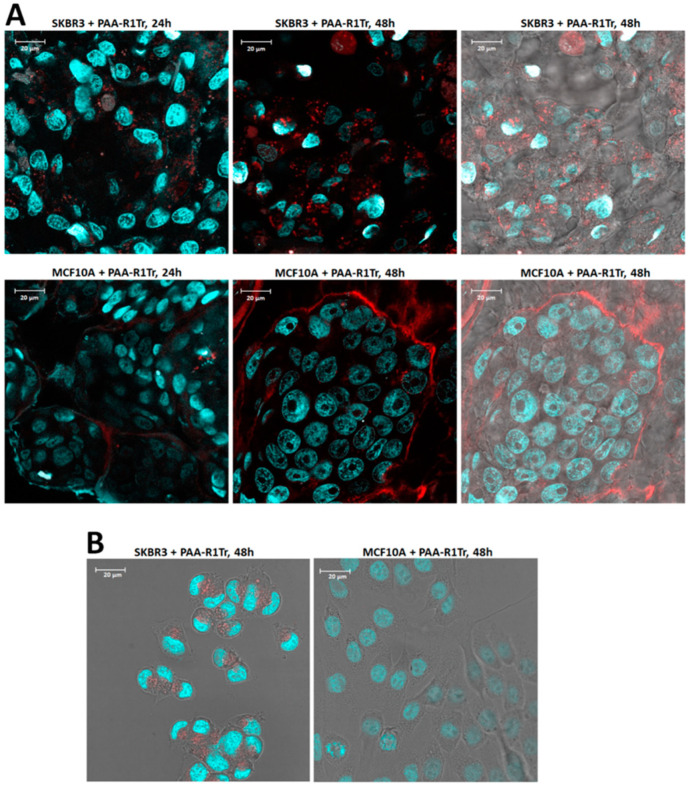
Panel (**A**) Images of SKBR3 spheroids (top row) and MCF10A spheroids (bottom row) incubated with truncated SKBR3-R1Tr-AF594 aptamers on polyacrylamide (1:225). Spheroids in the images on the left were incubated for 24 h with aptamer polymers and spheroids in the middle images for 48 h. The images on the right include the bright field images of the 48 h incubations. Panel (**B**) Images of SKBR3 cells (left) and MCF10A cells (right) grown in 2D in chamber slides, incubated with truncated SKBR3-R1Tr-AF594 aptamers on polyacrylamide (1:225). Cells were incubated for 48 h with aptamers and images were acquired at 100× magnification.

**Figure 7:**
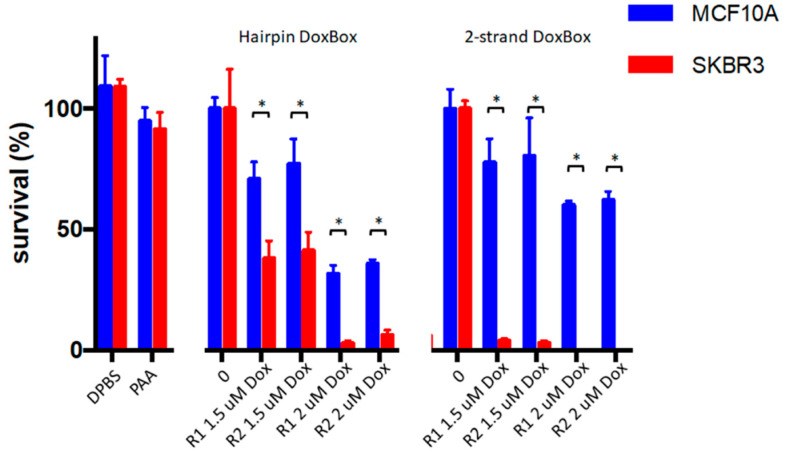
Cell viability of SKBR3 and MCF10A spheroids after 8 days of incubation with polymeric SKBR3-R1Tr or SKBR3-R2Tr aptamers and Dox boxes charged with doxorubicin. The text below the bars indicates the aptamer used and the Dox load, PAA indicates cells treated with multimeric aptamer polymers, conjugated to polyacrylamide. Cell viability was calculated relative to untreated spheroids (DBPS). * Statistical significant difference between MCF10A and SKBR3 (*p* < 0.01).

First, binding and internalization of the polymeric aptamers was assessed. As observed for free aptamers, polymers functionalized with SKBR3-R1Tr (AA-SKBR3-R1Tr) readily internalize after 24 h in SKBR3 cells grown as 3D spheroids or as 2D tissue and do not internalize into MCF10A cells (Figure 6). Next, the potential toxicity of free and multimeric aptamers was investigated, as this can be a major concern in designing therapeutic agents. Viability tests were conducted using a CellTiter Glo-3D^®^ viability assay. Neither the free aptamers nor the polymeric aptamers with unloaded Dox boxes induced cytotoxicity after 8 days of incubation, as shown in Figure 7. 

**Figure 8:**
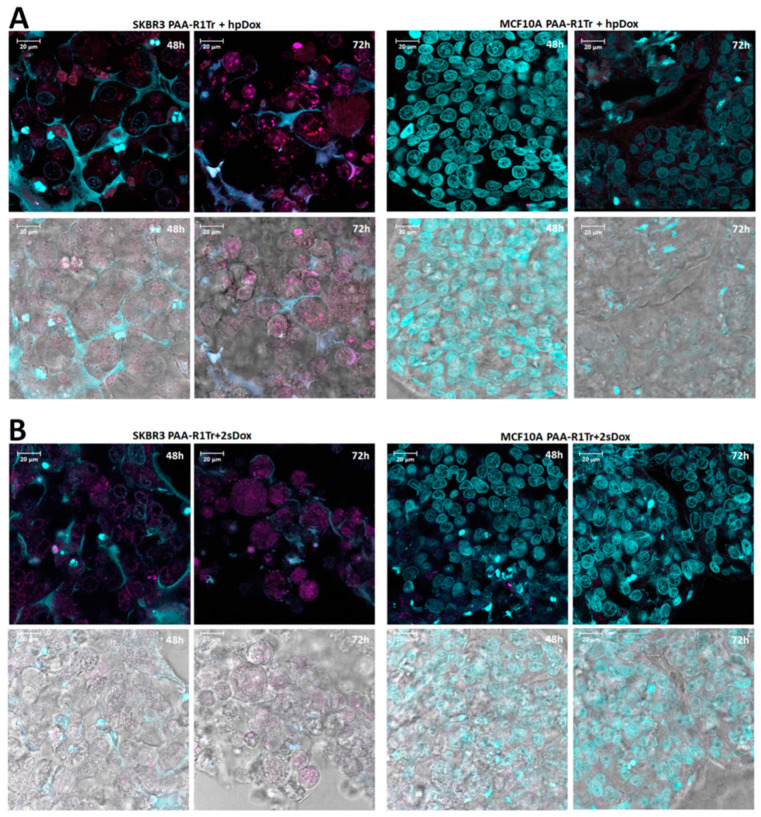
Images of SKBR3 spheroids (left half of each image set) and MCF10A spheroids (right half of each image set) incubated with polymeric SKBR3-R1Tr aptamers and Dox loaded Dox boxes (see Figure 2 and main text) for, respectively, 48 and 72 h. Panel (**A**) SKBR3-R1Tr + hairpin Dox box (hpDox) of which 1 in every 10 Dox boxes is Alexa Fluor 594 labeled. Panel (**B**) SKBR3-R1Tr + 2-strand Dox box (2sDox). The bottom row of each panel is the bright field image of the fluorescent image above.

Third, we assessed Dox toxicity by adding free Dox directly to SKBR3 and MCF10A spheroids at increasing Dox concentrations (Supplementary Figures S7 and S8). Increased toxicity is observed with increasing concentrations of Dox up to 2 µM after 8 days of incubation for both the target SKBR3 and control MCF10A spheroids. At a concentration of 2 µM free Dox, the survival rate levels off to 1–2% (Supplementary Figure S8). This concentration was used to investigate the effect of targeted delivery of Dox using polymeric aptamers. Loading of Dox in stoichiometric amounts to multimeric constructs with either hairpin or double stranded Dox boxes was determined using a fluorescence assay (Supplementary Figure S9). Targeted delivery of 2 µM Dox, contained within the polymeric constructs, was tuned by polymer concentration. Targeted delivery and specific cell death using the polymeric constructs was achieved for all polymeric constructs tested (Figure 7). The highest specificity and efficacy in targeted cell death was observed for the polymeric constructs containing the double stranded Dox boxes (Figure 7). The specific cytotoxic effect on SKBR3 spheroids is also clearly illustrated by confocal microscope images, which show disrupted SKBR3 cells and healthy, unaffected MCF10A cells after 2–3 days of incubation (Figure 8).

**Figure 9:**
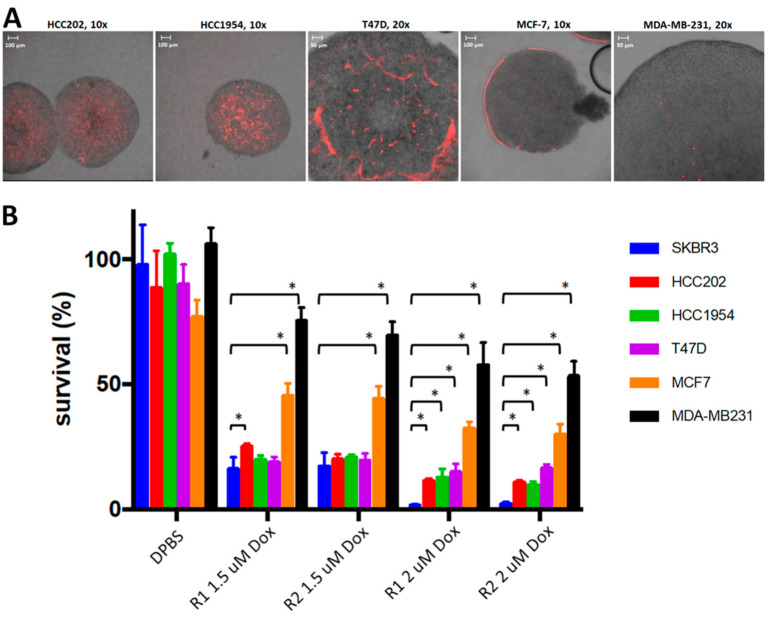
(**A**) Images of spheroids of various cell lines bound with full SKBR3-R1 aptamers labeled with Alexa Fluor 594. (**B**) Cell viability of spheroids of various cell lines after 8 days of incubation with polymeric SKBR3-R1Tr or SKBR3-R2Tr truncated aptamers and Dox loaded 2-strand Dox boxes. The text below the bars indicates the used aptamer and the Dox load. Cell viabilities were calculated relative to untreated spheroids (not shown in graph). * Pairwise statistical significant difference in treatment response compared to SKBR3 (*p* < 0.01).

Finally, we assessed therapeutic specificity by targeting polymeric aptamers with loaded Dox boxes to a number of different breast cancer cell lines representing different subgroups (Figure 9). Of these, the SKBR3 cells, HCC202, and HCC1954 are HER2+, T47D, and MCF7 are Luminal A (ER+, PR+, HER2-), and MDA-MB231 is triple negative (TNBC). As Figure 9 shows, the highest efficacy in cell death is achieved for the target SKBR3 cells. High efficacy is also observed for the other two breast cancer cell lines that overexpress HER2, i.e., HCC202 and HCC1954. MDA-MB-231, which is negative in ER, PR, and HER2 is the least affected by incubation with Dox loaded polymers, but still has a viability of only 50–80%, depending on Dox box type and Dox doses. Of the two luminal A cell lines, T47D shows a response as high as the HER2+ cell lines, while MCF7 shows a response in between the HER2+ and triple negative cell line (30–45% viability). HER2 is highly overexpressed on the surface of SKBR3 cancer cells [38,39], and would therefore be considered a likely candidate for the specific binding target of our raised aptamers. However, these results, in particular the observed high response of T47D and MCF7, suggest that the binding target for the selected aptamers is some other receptor, which after aptamer binding mediates internalization of the polymeric constructs, containing payloads of Dox. 

### 2.5. Imaging of Breast Cancer Tissue with Aptamers

**Figure 10:**
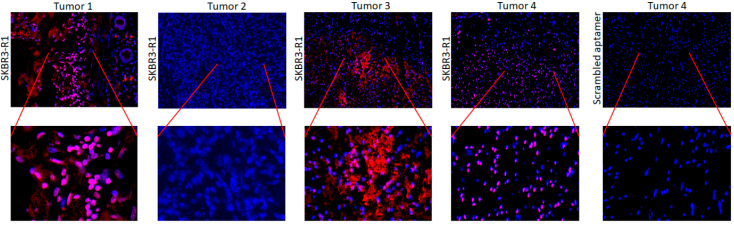
Fluorescent images of 4 FFPE breast cancer tissue sections stained with SKBR3-R1-AF594 or Scrambled-AF594 aptamer.

To assess the applicability of these aptamers in breast cancer diagnosis and their specificity we selected 4 formalin-fixed paraffin embedded breast cancer tissues and stained these with SKBR3-R1 and control scrambled aptamer (Figure 10). Whereas one tissue was negative, the other showed specific (peri) nuclear staining of breast cancer cells suggesting the aptamer is specific for particular cancers. 

## 3. Discussion

Several aptamers have previously been generated in the context of breast cancer, using peptides or purified receptor proteins [40,41,42,43,44,45,46,47,48,49,50,51,52], receptors overexpressed on engineered cell lines [53,54,55], or breast cancer cell lines as targets [56,57,58,59,60,61,62,63,64]. However, none of these aptamers have been selected using live 3D spheroids as targets. In this study, we used 3D spheroids of SKBR3 breast cancer cells as targets for aptamer selection, as these are a better mimic for the natural environment of solid tumors than the commonly used 2D tissue cultures, and should thus serve as a better model for selecting aptamers directed at breast cancer cells in vivo [11,12]. Using spheroids as targets, we were able to select DNA aptamers that bind SKBR3 cells with high affinity and specificity and display higher affinity for SKBR3 cells, grown as 3D spheroids than SKBR3 cells, grown as 2D monolayers. A few studies have been reported earlier in which aptamers were selected against SKBR3 cancer cells by Cell-SELEX [59,62]. However, in these studies, 2D tissue cultures were used as targets for selection and the aptamers were primarily used for binding studies and molecular subtyping of breast cancer. Neither report demonstrated therapeutic value of the selected aptamers. 

The concept of multivalency, where multiple ligands bind simultaneously to multiple targets, in particular to receptors on a cell surface, has been used for enhancing binding affinity of aptamers in different contexts and designs [64,65,66,67]. Various types of constructs have been designed to achieve multivalency: Multimeric constructs, connected with nucleic acid, peptide, or artificial linkers [37,48,68,69,70,71,72,73] of predefined length to tune the spacing in between aptamers, or nanoparticles [35,43,74,75,76,77,78,79] or liposomes [62,64,80,81,82] decorated with aptamers with pre-defined density. Polymeric designs as we report here have not been used widely in the construction and application of multimeric aptamers [37,70,71,72,73]. This design has several advantages. The spacing and valency of the aptamers can be easily tuned using appropriate monomeric ratios during the polymerization step. Moreover, the design allows for convenient incorporation of small DNA units for functionalization, e.g., with fluorescent dyes, or as drug carriers for specific treatment. This is far more efficient in synthesis and functionalization than extending selected aptamers for subsequent functionalization. Importantly, the polymeric constructs without loaded Dox did not confer cytotoxicity to the control cells, which is critical for using the polymeric constructs as a vehicle for therapeutics. Although we have not yet explored the added value of multivalency in affinity and specificity, this is likely to occur and can be a topic to be addressed in a next step of this research.

Due to the serious side effects of systemic chemotherapy, the targeted delivery of drugs using aptamers as vehicle by covalent conjugation of aptamers with small molecule anti-cancer drugs has been a major research topic in the aptamer field [7,8]. By taking advantage of the ability of doxorubicin to intercalate into CG-rich sequences (20-22) combined with our modular design, we created an efficient platform for drug delivery without the need for chemical conjugation. The results show that the polymeric aptamers with Dox boxes are selectively delivered to breast cancer cells and internalized, after which Dox accumulates in the nucleus. Treatment with these polymers carrying payloads of doxorubicin results in breast cancer cell death only, while non-malignant breast cells largely survive. Compared to earlier studies [25,26,27,28,29,30,31,32,33,34,35,47,64,71,75,78,79,81,82], the response to selective treatment with doxorubicin is high, resulting in <5% cell survival of SKBR3 spheroid cells at a dose of 2 µM. Taken together, these results illustrate the high potential of our multimeric approach in breast cancer therapy.

## 4. Materials and Methods

### 4.1. Cell lines and Cultures

SKBR3, T47D, MCF7, and MDA-MB231 cell lines were obtained from LCG Promochem (London, UK). MCF10A cells were obtained from LCG Promochem (London, UK). HCC1954 and HCC202 were a kind gift from John Martens (Erasmus MC, Rotterdam, The Netherlands). Cells were cultured at 37 °C, 5% CO_2_ in Dulbecco’s modified Eagles medium, high glucose, Glutamax supplemented with 10% (*v*/*v*) fetal bovine serum, 1 mM sodium Pyruvate, 1x non-essential amino acids, and 50 Units penicillin, 50 µg streptomycin (all from Gibco, ThermoFisher Scientific, Landsmeer, The Netherlands). MCF10A cells were cultured in Dulbecco’s modified Eagles medium, Nutrient Mixture F-12 (DMEM/F-12), supplemented with 5% Horse serum, 10 Units penicillin, 10 µg streptomycin (Gibco), 10 µg human EGF, 5 mg Insulin, 0.05 mg Cholera Toxin, 1 mM Dexamethasone (all from Sigma-Aldrich, Zwijndrecht, The Netherlands).

### 4.2. Spheroid Preparation 

Multicellular tumor spheroids were prepared from conventional monolayer cultures using the adaptive overlay technique [10]. Briefly, 10,000 cells were added per well in ultra-low attachment 96-well plates (Corning, Corning, NY, USA) in 100 µL standard culture medium with 2.5% Matrigel (BD Biosciences, San Jose, CA, USA). After a centrifugation step at room temperature (10 min at 1000× *g*) 100 µL culture medium was added and cells were allowed to form spheroids for 5 days. 

### 4.3. Selection Procedure

The random DNA library and all other oligonucleotides (Supplementary Table S1) were purchased from IDT (Leuven, Belgium) and purified in house using denaturing PAGE. The Cell-SELEX procedure was carried out as described by Sefah et al. [83] with minor modifications. In the first two rounds of selection, 5 million SKBR3 cells grown to confluency were used as positive target cells and 10 million MCF10A cells for negative selection. From the third round on, 1 million SKBR3 cells grown in spheroids were used for the positive selection and further negative selections were omitted. The binding stringency in each round of selection was increased by lowering the amount of DNA for binding, shortening the time of binding, and increasing the percentage of fetal bovine serum (FBS), the washing time, and washing volume (Supplementary Table S2). Since the cells were grown in spheroids, FACS analysis was not readily applicable to monitor the selection progress and selection was therefore continued blindly for a total of 12 rounds. The adapter sequences of the DNAs obtained after each selection round were extended by PCR with barcoded primers suitable for multiplexed next generation sequencing (Supplementary Table S1) and native PAGE purified PCR products were sequenced on an Illumina HiSeq2000 platform (ServiceXS, Leiden, The Netherlands). Raw sequencing data was processed and analyzed using the public data server at www.usegalaxy.org (accessed on 3 February 2017) [84].

### 4.4. Fluorescent Labeling of Truncated Aptamer DNAs

DNA aptamers for labeling purposes were purchased from IDT with a 3’-terminal ribose. The terminal 3’-ribonucleotides of the aptamer DNAs were oxidized by incubating 10 μM of DNA in 100 mM NaAc (pH 5.2) containing 250 μM freshly prepared NaIO_4_ for 30 min on ice. After 2-fold dilution of the reaction mixture and adjustment of the NaAc (pH 5.2) concentration to 300 mM, the DNA was precipitated with 2-propanol and the pellet washed with ice-cold 70% ethanol and subsequently air dried. The DNA was dissolved to 10 μM in 100 mM NaAc (pH 5.2) containing 100 μM Alexa Fluor hydrazide 594 and incubated for 60 h at 4 °C in the dark. The DNA was subsequently purified from the uncoupled label by precipitation with 2-propanol and thorough rinsing of the pellet with ice-cold 70% ethanol. The full-length aptamer DNAs were first equipped with a 3’-ribonucleotide by ligating a 13-nt oligonucleotide, representing the terminal part of the 3’-adapter sequence to the rest of the aptamer because of sequence length restrictions of the commercially available oligonucleotides. Ligations were carried out at 10 μM of a stoichiometric DNA assembly that was heat annealed prior to the addition of T4 DNA ligase and incubated for 3 h at 37 °C. Ligation products were purified using denaturing PAGE prior to subjecting the DNAs to the fluorescent labeling procedure. All fluorescent labeling yields were 85–95% as determined by UV-VIS spectroscopy using a Nanodrop spectrophotometer (IsogenLife Science, De Meern, The Netherlands).

### 4.5. Co-Polymerisation of DNA with Acrylamide

5’-Acrydite modified truncated aptamers were co-polymerized with acrylamide in a molar ratio of 1:225 (DNA: acrylamide) alone or in combination with hairpin Dox boxes (hpDox) or 2-strand Dox box lead strand (2sDox-lead) in a ratio of 1:2.5:225 (DNA:Dox:acrylamide). For microscopic imaging experiments, an Alexa Fluor 594 (AF594) modified aptamer (ratio 1:5) or hairpin Dox box (ratio 1:10) was co-polymerized. In general, 10 nmol of aptamer DNA was dried in a Speed-Vac (Thermo-scientific), either alone or mixed with 22.5 nmol of hpDox or 2sDox-lead. The dried DNA was dissolved in 16 μL of a freshly prepared solution of 1% acrylamide (140 mM) in Dulbecco’s PBS (DPBS), mixed well and supplemented with 0.5 μL of freshly prepared solutions of 10% APS and 10% TEMED. Polymerization was carried out overnight at 25 °C, 500 rpm in a Thermoshaker (Eppendorf). The next day, extra portions of TEMED and APS were added and polymerization was continued for 5 h at 37 °C, 500 rpm in a Thermoshaker. The volume was adjusted with DPBS to 500 μL resulting in a 20 μM solution with respect to the aptamer DNA. The degree of co-polymerization was ≥90% as confirmed by visualization on denaturing PAGE (Supplementary Figure S10).

### 4.6. Binding, Internalization, and Doxorubicin Loading Capacity Assays 

For binding assays, stock solutions of free and/or polymeric DNA aptamer were prepared by snap-cooling a boiling (95 °C) solution of 5 μM aptamer DNA in DPBS with 5 mM MgCl2 (DPBS-Mg) on ice water. Three spheroids were carefully pipetted out of the 96-well plate using end-cut 200 μL filter tips and pooled together in a microcentrifuge tube and allowed to settle on the bottom. Supernatant was carefully pipetted off and the spheroids were washed twice with 200 μL of binding buffer (DPBS, 5 mM MgCl_2_, 22.5 mM d-glucose, 1 mg/mL BSA, and 100 μg/mL tRNA (Baker’s Yeast)). 100 μL of 50 nM aptamer solution in binding buffer was added to the spheroids and incubated for 1 h at room temperature with occasional swirling of the spheroids, taking care not to disturb the spheroid structure. Spheroids were washed once with 200 μL of binding buffer and then incubated for 20 min in 200 μL of 1 μg/mL Hoechst 33342 in binding buffer for nuclei staining. Spheroids were transferred to an epoxy resin masked 6-well microscopy slide (Marienfeld, #1215130). Excess of binding buffer was pipetted off and spheroids were included with Fluoromount G (Invitrogen, Carlsbad, CA, USA) under a coverslip. For internalization and doxorubicin loading capacity assays, stock solutions of free and/or polymeric DNA aptamers (with or without Dox Boxes) were prepared in DPBS-Mg at 1 μM with respect to the aptamer DNA concentration. In case of the 2-strand Dox box, a stoichiometric amount of 2sDox-lag (complementary to 2sDox-lead) was added prior to heat-annealing. When required for the experiment, doxorubicin was added to the desired concentration after heat-annealing of the DNAs from a freshly prepared 100 μM solution in DPBS-Mg produced out of a 5 mM DMSO stock. For internalization assays, (polymeric) DNA aptamers were added at 100 nM to the growth medium of 2D cell cultures grown in 8-well chamber slides and/or to the growth medium of spheroids in 96-well plates and incubated for 24, 48, and/or 72 h. Spheroids were then harvested, washed, and stained with Hoechst prior to inclusion on microscopy slides as described above. 

### 4.7. Confocal Laser Scanning Microscopy

All microscopy experiments were carried out on a Sp8x confocal laser scanning microscope equipped with a 405 nm diode laser and a pulsed white light laser (Leica microsystems) using 10× Air, 20× Air, and 100× Oil magnification objectives. For the binding assays and the 2D internalization assay, microscope settings were: 50% laser power, 6% shutter intensity for the 405 nm laser line, and 5% for the 594 nm laser line, respectively. Images were acquired consecutively to prevent cross talk between dye excitations or bleeding of emissions in different channels. The scan speed was 200 Hz and the fluorescent signal was visualized using hybrid detectors in BrightR mode, with line accumulation and frame averaging set to three. Settings for the 3D internalization assays were identical, but the shutter intensities were 3% for the 405 nm laser line and 10% for the 594 nm laser line, or 485 nm laser line for doxorubicin excitation. Images were acquired and processed using LasX software, version 1.8.1.13759 (Leica, Wetzlar, Germany). 

### 4.8. In Vitro Cytotoxicity Assays

Polymeric aptamers co-polymerized with Dox boxes were prepared and loaded with doxorubicin to the desired concentrations as described above. The growth medium of 8-days old spheroids was supplemented with polymeric aptamers to 100 nM with respect to the DNA aptamer (250 nM Dox box) in sextuple and incubated for another 8 days at 37 °C, 5% CO_2_ in the incubator stove. On day four, 50% (100 μL) of the medium was replaced by fresh medium and an extra dose of doxorubicin loaded polymeric aptamers was added to 50 nM. After 8 days, spheroids were individually transferred in 100 μL of medium to a luminescence plate (Thermo Scientific, #9502887) and mixed thoroughly with 100 μL of CellTiter-Glo^®^ 3D (Promega, Madison, WI, USA). This was incubated for 45 min at 70 rpm on an orbital shaking platform at room temperature in the dark, and the chemiluminescence was read in a Victor3 plate reader (PerkinElmer, Waltham, MA, USA). Similarly, doxorubicin titration curves were generated for SKBR3 and MCF10A spheroids to determine the optimal doxorubicin dose.

### 4.9. Preparation and Imaging of Breast Cancer Tissue

Paraffin embedded fixated breast cancer tissue was cut into 5-micron sections. Sections were dewaxed, rehydrated, and washed with demineralized water and subsequently incubated in citrate buffer (pH 6.0) at 96 °C for 30 min (HIER—heat induced epitope retrieval), allowed to cool down to room temperature, and then rinsed with demineralized water. Sections were pre-incubated with binding buffer (DPBS-Mg + 4.5 g/L d-glucose) for 30 min at room temperature. 200 μL of 100 nM SKBR3R1-AF594 or Scramble-AF594 in binding buffer was added to the sections and incubated for 1 h at room temperature. Sections were rinsed with binding buffer, counterstained with Hoechst 33342 (nuclear staining) for 5 min at room temperature, and coverslipped with Fluoromount. Images were acquired using Ivision-Mac on a Zeiss AXIO Scope A1.

### 4.10. Statistical Analysis 

Data were analyzed using GraphPad Prism 5 and are shown as mean + standard deviation. Statistical significance of differences between 2 groups was assessed using Student’s t-tests, with discovery determined using the two-stage linear step-up procedure of Benjamini, Krieger, and Yekutieli [85], with Q = 1%. Differences between more than 2 groups were established using one-way ANOVA with Bonferroni corrected post hoc tests. Statistical significance was assumed a priori at α = 0.01. 

## 5. Conclusions

In summary, we have developed an innovative modular DNA aptamer platform based on 3D cell- SELEX and polymer multivalency. We successfully selected DNA aptamers with high affinity by targeting spheroids of SKBR3 breast cancer cell lines. By copolymerization of selected DNA aptamers with Dox containers efficient vehicles with high loading capacity were constructed for targeted drug delivery. Fluorescence imaging and viability tests showed high specificity and efficacy of the polymeric DNA aptamer platforms. Most importantly, targeted delivery of doxorubicin using multivalent aptamer-Dox box polymers to breast cancer spheroids resulted in high selective toxicity. Given the high affinity, selectivity, and cytotoxic efficacy, the polymeric modular platform holds great promise for diagnostics and treatment of breast cancer.

## Figures and Tables

**Table 1:** Top 10 of raised aptamer sequences after 12 rounds of selection.

Name/Rank	Aptamer Sequence ^1^	Enrichment in Round 12
**SKBR3-R1**	ATCCAGAGTGACGCAGCATCGTTGCTAATAGTGCCGACGCCGGCGAAATTAATAGGTCGGTCACTTCTCCTGTAGGCACCATCAATC	22.9%
**SKBR3-R2**	ATCCAGAGTGACGCAGCAAGGTTTCCAACCGAAGGGCGCAAGGCGAAGCCGTGGGGTTGCAAACCGCGAACATCTGTAGGCACCATCAATC	8.17%
**SKBR3-R3**	ATCCAGAGTGACGCAGCACCAGATGCGAGTTCACTCGGTAGGGCGAAGCCGTGGAATTAATCGTTCGCCCTAACTGTAGGCACCATCAATC	3.75%
**SKBR3-R4**	ATCCAGAGTGACGCAGCACGGACAACCTCTCGTCGTGATTGGGCGAAGCCGTGGAATTAATCGTTCGCCCGCACTGTAGGCACCATCAATC	3.64%
**SKBR3-R5**	ATCCAGAGTGACGCAGCAGCAGGCACGTGGGTGATCACATGGGCGAAGCCGTGGAATTAATCGTTCGTCCATTCTGTAGGCACCATCAATC	1.55%
**SKBR3-R6**	ATCCAGAGTGACGCAGCAAATATACATAGCCCTAGCAGTGAGGCGAAGCCGTGGAATTAATCGTTCGCCGTGACTGTAGGCACCATCAATC	1.17%
**SKBR3-R7**	ATCCAGAGTGACGCAGCAGTGTAAACAGCTCGACGTCCCGGGGCGAAGCCGTCCGGCCTCTACTTAATTCGCGCTGTAGGCACCATCAATC	1.05%
**SKBR3-R8**	ATCCAGAGTGACGCAGCAACTCCTGGAACTGTCCCTAAGCGGGCGAAGCCGTGGAATTAATCGTTCGCCACTGCTGTAGGCACCATCAATC	1.04%
**SKBR3-R9**	ATCCAGAGTGACGCAGCATCTACACCAGTGTTTTAAGTTGAGGCGAAGCCGTGGAATTAATCGTTCGCCTCCGCTGTAGGCACCATCAATC	0.9%
**SKBR3-R10**	ATCCAGAGTGACGCAGCACTTATGTCTTCTTCCATGTGTGTGGCGAAGCCGTGGAATTAATCGTTCGCCACATCTGTAGGCACCATCAATC	0.59%

^1^ Sequences in red indicate the constant adapter regions for amplification and the internal hairpin.

## Data Availability

The data presented in this study are available on request from the corresponding authors.

## Supplementary Materials

The following are available online at https://www.mdpi.com/article/10.3390/ph14040349/s1. Table S1: Overview of used oligonucleotides, Table S2: Summary of the aptamer selection procedure, Table S3: Abundance and alignment of the top 10 DNA aptamers, Table S4: Percentages of enrichment of the top 5 DNA aptamers in the 12 rounds of selection, Figure S1: Secondary structure models of the top 10 DNA aptamers, Figure S2: Z-stacks of SKBR3 and MCF10A spheroids, incubated with full SKBR3-R1 aptamer, Figure S3: Images of SKBR3 and MCF10A spheroids, incubated with randomized aptamer (scramble-AF594), Figure S4: Binding curve of aptamer SKBR3-R1Tr to SKBR3 spheroids, Figure S5: Internalization experiment of SKBR3 and MCF10A spheroids with randomized aptamer, Figure S6: Mechanism of aptamer internalization, Figure S7: Images of SKBR3 and MCF10A spheroids with free Doxorubicin, Figure S8: Effect of free Doxorubicin concentrations on the viability of SKBR3 and MCF10A spheroids, Figure S9: Determination of the doxorubicin load of polymeric aptamer constructs with and without hairpin Dox boxes and 2-strand Dox boxes, Figure S10: Quality check of co-polymerization of polymeric constructs.
