# Supplementary material for: Improving Breast Cancer Treatment Specificity Using Aptamers Obtained by 3D Cell-SELEX
Source: Pharmaceuticals (Basel). 2021 Apr 9;14(4):349. doi: 10.3390/ph14040349 (PMC8068899; doi:10.3390/ph14040349)
Supplement: Supplementary file 1 [file pharmaceuticals-14-00349-s001.pdf]

Supplementary Information

DNA	Sequence	Usage
Selection stage		
random library	ATCCAGAGTGACGCAGCA-$N_{(23)}$-GGCGAAGCC-$N_{(23)}$-CATCTGTAGGCACCATCAATC	Selection
library primer fwd	FAM-ATCCAGAGTGACGCAGCA	Library amplification
library primer rvd	Biotin-GATTGATGGTGCCTACAG	Library amplification
Next generation sequencing		
Library primers (6)	AATGATACGGCGACCACCGAGATCTACACTCTTTCCCTACACGACGCTCTTCCGATCT-$N_{(0-5)}$-ATCCAGAGTGACGCAGCA	NGS
Indexing primers (12)	CAAGCAGAAGACGGCATACGAGAT<u>******</u>GTGACTGGAGTTCAGACGTGTGCTCTTCCGATCT-$N_{(0-5)}$-GATTGATGGTGCCTACAG	NGS
Used barcodes on indexing primers	AGTCGT TCGGAT ATGCTG ACGTAC GACGTC GTCGTA GGTCAG CGTACT CACTGC CTAACA TCTAGG TGATGA	Multiplexing
Aptamer labeling for imaging		
SKBR3-R1Tr_Mic	CAGCATCGTTGCTAATAGTGCCGACGCCGGCGAAATTAATAGGTCGGTCACrU	Imaging
SKBR3-R2Tr_Mic	GCTTTCCAACCGAAGGGCGCAAGGCGAAGCCGTGGGGTTGCAAAGrC	Imaging
SKBR3-R1full_p1	ATCCAGAGTGACGCAGCATCGTTGCTAATAGTGCCGACGCCGGCGAAATTAATAGGTCGGTCACTTCTCCTGTA	Ligation
SKBR3-R2full_p1	ATCCAGAGTGACGCAGCAAGGTTTCCAACCGAAGGGCGCAAGGCGAAGCCGTGGGGTTGCAAACCGCGAACATCTGTA	Ligation
SKBR3-R1scramble_p1	ATCCAGAGTGACGCAGCACTAATATCGTTGACGCCGGTGCCGTTAATAGCGAAATCACTTGGTCGGCTCCTGTA	Ligation
SKBR3-R1_p_splint	GATGGTGCCTACAGGAGAAGTGAC	Ligation
SKBR3-R2_p_splint	GATGGTGCCTACAGATGTTCGCGG	Ligation
SKBR3-R1scramble_p_splint	GATGGTGCCTACAGGAGCCGACCA	Ligation
SKBR3-R1/2/scramble_p2	Phos-GGCACCATCAATrC	Ligation
Aptamer copolymerization (and labeling for imaging)		
SKBR3-R1Tr	Acry-TTTTTTCAGCATCGTTGCTAATAGTGCCGACGCCGGCGAAATTAATAGGTCGGTCAC(T/rU)	Cell viability/imaging
SKBR3-R2Tr	Acry-TTTTGACGCAGCAAGGTTTCCAACCGAAGGGCGCAAGGCGAAGCCGTGGGGTTGCAAACCGCGAA	Cell viability/imaging
Hairpin DoxBox	Acry-TTTTTACGACGACGACGACGACGACTTGTCGTCGTCGTCGTCGTT(T/rU)	Dox carrier/imaging
2-strand DoxBox lead	Acry-TTTTTCGACGACGACGACGACGACGACGA	Dox carrier
2-strand DoxBox lag	TCGTCGTCGTCGTCGTCGTCGTCG	Dox carrier

Supplementary Table 1. Overview of used oligonucleotides. The forward library primer is modified with a 5/6-FAM fluorophore and the reverse library primer with a biotin group for strand separation during purification. The $N_{(0-5)}$ in the NGS library and indexing primers denotes random nucleotides that are present in the primers, incremented by one N-residue per two rounds of selection to generate sequence complexity during next generation sequencing. The asterisks in the indexing primers indicate the position of the barcodes.

Round	ssDNA for selection (pmol)	Cells (x10^6), growth type	Binding (min)	FBS (%)	Washing (min)	Washing (ml) x 3	Eluate (ml), eluent	PCR (ml), cycles (No.)	Yield ssDNA (pmol)
**1**	25000	5, confluent	60	---	---	10	3, Milli-Q	6.0, 10	1150
**2**	1150	5, confluent	60	---	---	10	1.4, BB	5.0, 14	617.5
**3**	615	1, spheroid	60	---	---	1.5	0.4, DPBS	2.0, 10	325
**4**	325	1, spheroid	60	---	5	5	0.6, DPBS	1.0, 10	135
**5**	135	1, spheroid	60	10	5	5	0.6, DPBS	3.5, 12	220
**6**	100	1, spheroid	60	12.5	5	5	0.75, DPBS	1.5, 14	365
**7**	90	1, spheroid	55	15	5	6	0.9, DPBS	1.5, 14	150
**8**	80	1, spheroid	50	17.5	5	7	0.9, DPBS	1.5, 14	240
**9**	70	1, spheroid	45	20	5	8	0.9, DPBS	1.5, 14	385
**10**	60	1, spheroid	40	20	10	9	0.9, DPBS	1.5, 14	395
**11**	50	1, spheroid	35	20	20	10	0.9, DPBS	1.5, 13	280
**12**	40	1, spheroid	30	20	30	15	0.9, DPBS	1.5, 14	340

Supplementary Table 2: Summary of the aptamer selection procedure. FBS: foetal bovine serum; Milli-Q: ultra-pure water (Millipore); BB: binding buffer (see main text); DPBS: Dulbecco's PBS. Washes were carried out three times for each selection round. PCRs were conducted in aliquots of 100 µl using in house purified *Pfu* DNA polymerase (Nelissen, NAR 2009)

N reads	Rank	Family
588	42	ATCCAGAGTGACGCAGCAACGTTGCTAATAGTGCCGACGCCGGCGAAATTAATAGGTCGGTCACTTCTCCTGTAGGCACCATCAATC
875	31	ATCCAGAGTGACGCAGCATCGTTTCTAATAGTGCCGACGCCGGCGAAATTAATAGGTCGGTCACTTCTCCTGTAGGCACCATCAATC
2068	22	ATCCAGAGTGACGCAGCATCGTTGCTAATAGTGCCGACGTCGGCGAAATTAATAGGTCGGTCACTTCTCCTGTAGGCACCATCAATC
2236	20	ATCCAGAGTGACGCAGCATCTTTGCTAATAGTGCCGACGCCGGCGAAATTAATAGGTCGGTCACTTCTCCTGTAGGCACCATCAATC
412795	1	ATCCAGAGTGACGCAGCATCGTTGCTAATAGTGCCGACGCCGGCGAAATTAATAGGTCGGTCACTTCTCCTGTAGGCACCATCAATC
237	91	ATCCAGAGTGACGCAGCAAGGTTTCCAACCGAAGGGCGCAAGGCGAAGCCGTGGGGTTGTAAACCGCGAACATCTGTAGGCACCATCAATC
247	85	ATCCAGAGTGACGCAGCAGGGTTTCCAACCGAAGGGCGCAAGGCGAAGCCGTGGGGTTGCAAACCGCGAACATCTGTAGGCACCATCAATC
247	84	ATCCAGAGTGACGCAGCAAGGTTTCCAACCGAAGGGCGCAAGGCGAAGCCGTGGGGTTGCAAATCGCGAACATCTGTAGGCACCATCAATC
325	73	ATCCAGAGTGACGCAGCAAGGTTTCCAACCGAAGGGCGCAAGGCGAAGCCGTGGGGTTGCAAACCGCTAACATCTGTAGGCACCATCAATC
462	58	ATCCAGAGTGACGCAGCACGGTTTCCAACCGAAGGGCGCAAGGCGAAGCCGTGGGGTTGCAAACCGCTAACATCTGTAGGCACCATCAATC
147248	2	ATCCAGAGTGACGCAGCAAGGTTTCCAACCGAAGGGCGCAAGGCGAAGCCGTGGGGTTGCAAACCGCGAACATCTGTAGGCACCATCAATC
572	43	ATCCAGAGTGACGCAGCACCAGATGCGAGTTCACTCGGTAGGGCGAAGCCGTGGAATTAATCGTTCGCCCGCACTGTAGGCACCATCAATC
67575	3	ATCCAGAGTGACGCAGCACCAGATGCGAGTTCACTCGGTAGGGCGAAGCCGTGGAATTAATCGTTCGCCCTAACTGTAGGCACCATCAATC
624	40	ATCCAGAGTGACGCAGCACGGACAACCTCTCGTCGTGATTGGGCGAAGCCGTGGAATTAATCGTTCGCCCTAACTGTAGGCACCATCAATC
65676	4	ATCCAGAGTGACGCAGCACGGACAACCTCTCGTCGTGATTGGGCGAAGCCGTGGAATTAATCGTTCGCCCGCACTGTAGGCACCATCAATC
854	32	ATCCAGAGTGACGCAGCAGCAGGCACGTTGGTGATCACATGGGCGAAGCCGTGGAATTAATCGTTCGTCCATTCTGTAGGCACCATCAATC
27903	5	ATCCAGAGTGACGCAGCAGCAGGCACGTGGGTGATCACATGGGCGAAGCCGTGGAATTAATCGTTCGTCCATTCTGTAGGCACCATCAATC
27903	6	ATCCAGAGTGACGCAGCAAATATACATAGCCCTAGCAGTGAGGCGAAGCCGTGGAATTAATCGTTCGCCGTGACTGTAGGCACCATCAATC
312	76	ATCCAGAGTGACGCAGCAGTGTAAACAGCTCGACGTCCCGGGGCGAAGCCGTCCGGCCTCTACTTAATTCGCACTGTAGGCACCATCAATC
18462	7	ATCCAGAGTGACGCAGCAGTGTAAACAGCTCGACGTCCCGGGGCGAAGCCGTCCGGCCTCTACTTAATTCGCGCTGTAGGCACCATCAATC
564	44	ATCCAGAGTGACGCAGCAACTCCTGGAACTGTCCCTAAGCTGGCGAAGCCGTGGAATTAATCGTTCGCCACTGCTGTAGGCACCATCAATC
18832	8	ATCCAGAGTGACGCAGCAACTCCTGGAACTGTCCCTAAGCGGGCGAAGCCGTGGAATTAATCGTTCGCCACTGCTGTAGGCACCATCAATC
16149	9	ATCCAGAGTGACGCAGCATCTACACCAGTGTTTTAAGTTGAGGCGAAGCCGTGGAATTAATCGTTCGCCTCCGCTGTAGGCACCATCAATC
10657	10	ATCCAGAGTGACGCAGCACTTATGTCTTCTTCCATGTGTGTGGCGAAGCCGTGGAATTAATCGTTCGCCACATCTGTAGGCACCATCAATC

Supplementary Table 3. Abundance and alignment of the top 10 DNA aptamers obtained by Cell - SELEX after the 12th round, grouped in families using ClustalX on the top 100 most frequent sequences. N reads represents the total number of reads for a specific sequence. Adapter sequences are in light blue. The total number of reads of the 12th round was 1,8 x 10^6.

Variable part of sequence (5'-->3')	Rank	Percentage of read in analyzed round											
		1	2	3	4	5	6	7	8	9	10	11	12
TCGTTGCTAATAGTGCCGACGCCGGCGAAATTAATAGGTCGGTCACTTCTC	1	0.22	0.28	0.28	0.48	0.72	0.52	1.86	8.10	8.40	14.37	22.31	22.90
AGGTTTCCAACCGAAGGGCGCAAGGCGAAGCCGTGGGGTTGCAAACCGCGAACAT	2	0.04	0.07	0.06	0.10	0.16	0.14	0.22	0.54	1.31	5.32	8.20	8.17
CCAGATGCGAGTTCACTCGGTAGGGCGAAGCCGTGGAATTAATCGTTCGCCCTAA	3	0.06	0.09	0.10	0.16	0.27	0.24	1.07	5.10	4.51	3.88	4.26	3.75
CGGACAACCTCTCGTCGTGATTGGGCGAAGCCGTGGAATTAATCGTTCGCCCGCA	4	0.07	0.10	0.12	0.18	0.33	0.30	1.45	6.30	4.25	4.05	3.56	3.64
GCAGGCACGTGGGTGATCACATGGGCGAAGCCGTGGAATTAATCGTTCGTCCATT	5	0.02	0.03	0.04	0.06	0.10	0.10	0.45	2.46	1.70	1.79	2.13	1.55
**Total number of analyzed reads in round (x10^6)**		**21.66**	**7.54**	**14.90**	**7.79**	**4.36**	**6.95**	**5.82**	**4.23**	**10.92**	**5.13**	**2.28**	**1.80**

Supplementary Table 4. Percentages of enrichment of the top 5 raised aptamer sequences from round 12 in each round of the selection procedure. Only the random sequences flanking the hairpin sequence in red are shown.

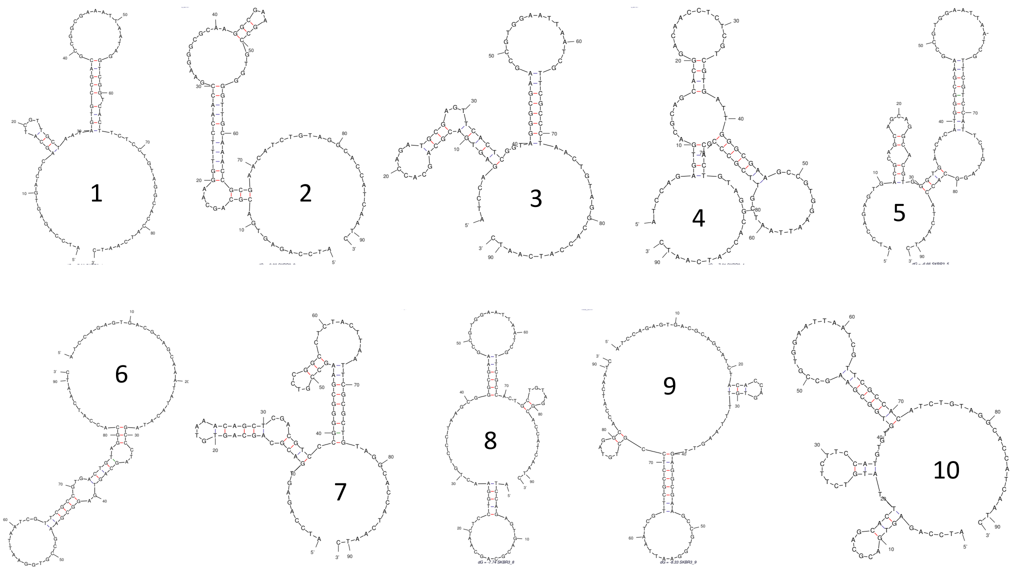

Supplementary Figure 1. Secondary structure models of the top 10 selected DNA aptamers. Folding simulations were carried out using the Mfold webserver (Zuker, NAR 2003) at a temperature of 37°C with ionic strength conditions of 150 mM NaCl and 5 mM $MgCl_2$.

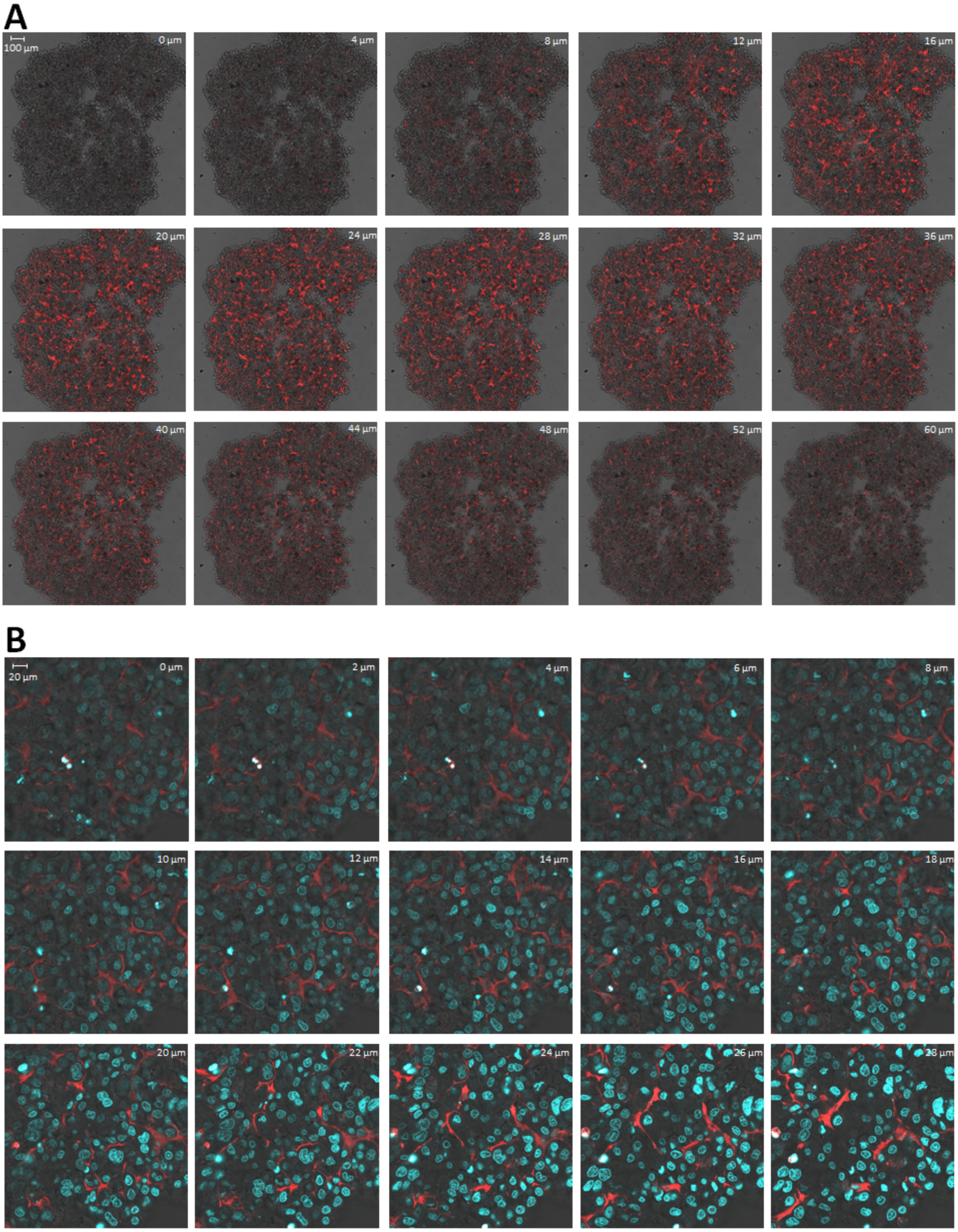

Supplementary Figure 2. Z-stacks (at 2 µm intervals) of SKBR3 (panel A) and MCF10A (panel B) spheroids incubated with full SKBR3-R1 aptamers labeled with Alexa Fluor 594 after one hour incubation at room temperature.

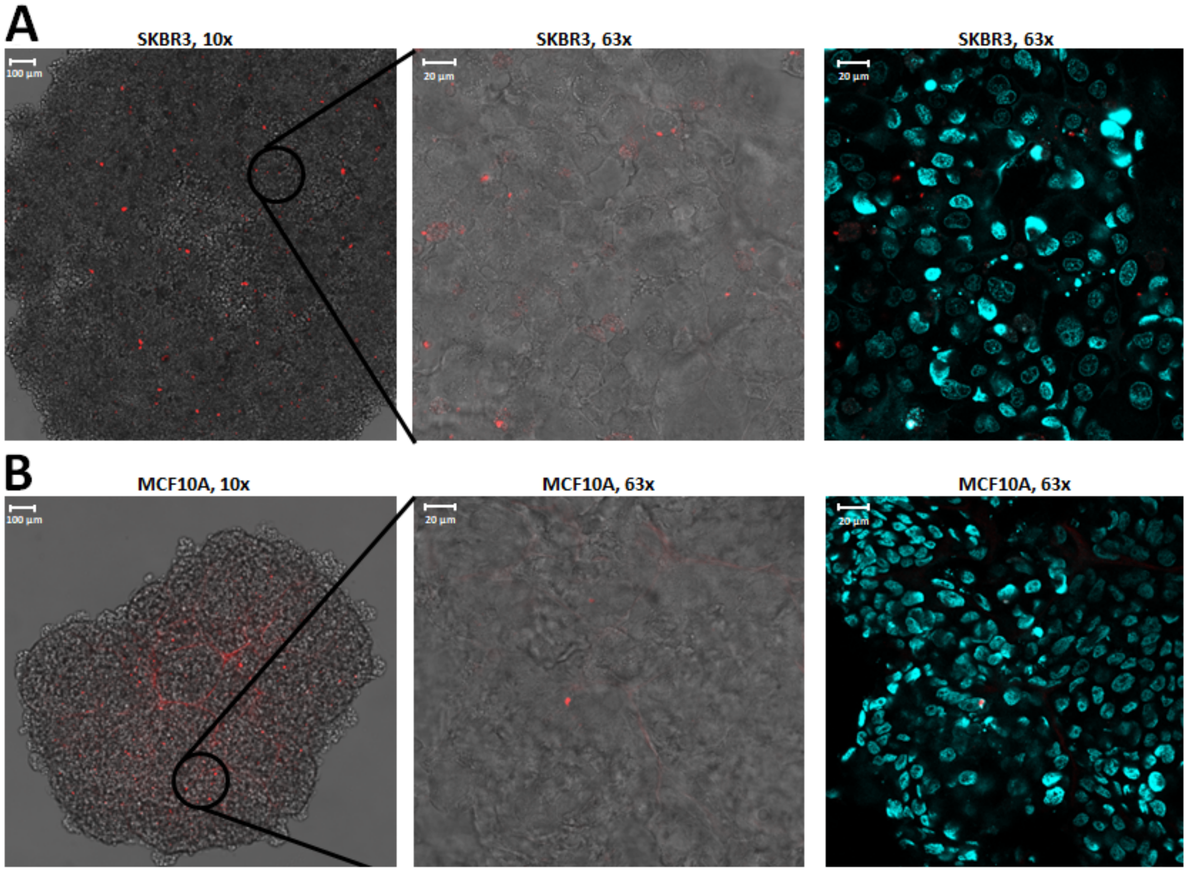

Supplementary Figure 3. Images of SKBR3 (panel A) and MCF10A (panel B) spheroids incubated with control, randomized full aptamer scramble-AF594 labeled with Alexa Fluor 594 after one-hour incubation at room temperature. Images on the left are at 10x, images in the middle at 63x magnification, bright field and AF594 signal (red). The images on the right show Hoechst-stained nuclei (cyan) and the AF594 signal (red). Panel A) SKBR3 spheroids. Panel B) MCF10A spheroids.

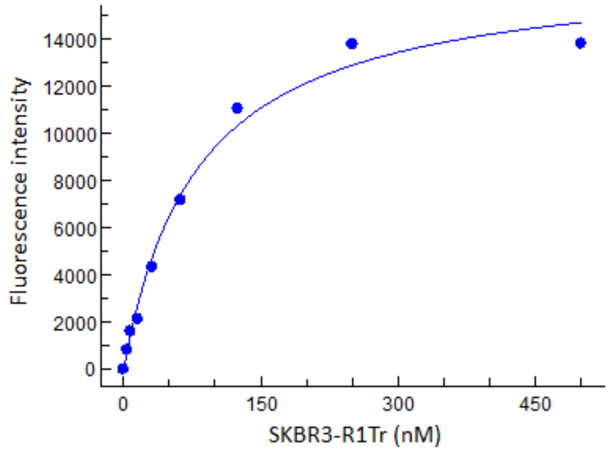

Supplementary Figure 4. Titration experiment of fluorescent labelled SKBR3-R1Tr to SKBR3 spheroids. Fitting of the binding curve reveals an apparent binding constant (Kd) of 81.4 nM.

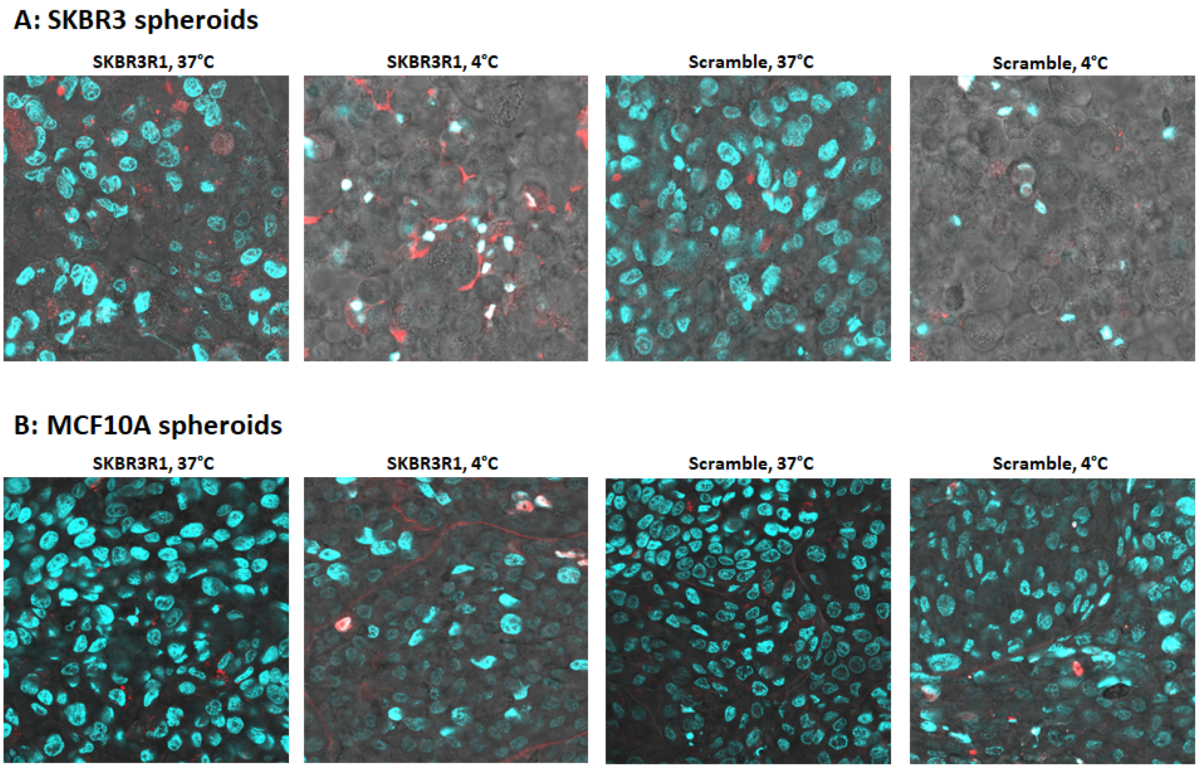

Supplementary Figure 5. Control aptamer Scramble does not internalize into SKBR3 or MCF10A spheroid cells. Punctate patterns of fluorescence spots, typical for internalization are only observed for SKBR3 spheroid cells, incubated at 37C with SKBR3R1 aptamers.

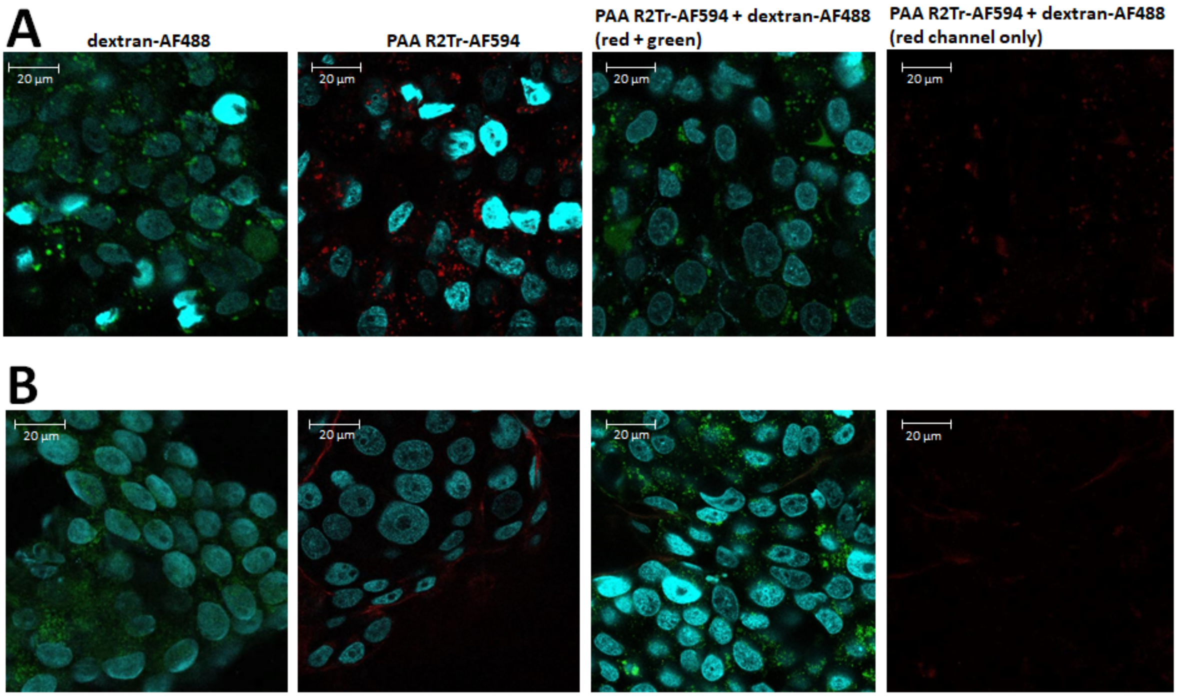

Supplementary Figure 6. Mechanism of aptamer uptake. Images of SKBR3 spheroids (upper panel A) and MCF10A spheroids (lower panel B) incubated with Alexa Fluor 488 modified Dextran, Alexa Fluor 594 modified polymeric SKBR3-R1Tr2, or combination hereof. Fluorescent-labelled dextran is taken up by the target SKBR3 cells (upper panel A) as well as control MCF10A (lower panel B) cells. Fluorescent-labelled aptamers mixed with fluorescent-labelled dextran are only taken up by SKBR3 and co-localize with the dextran label, indicating a macropinocytosis type of mechanism for internalization. Dextran was labeled via the same procedure as described in the methods section of the main text for the DNA aptamers and was additionally dialyzed in a 10 kDa MWCO Amicon ultrafiltration device (Merck-Millipore) prior to use.

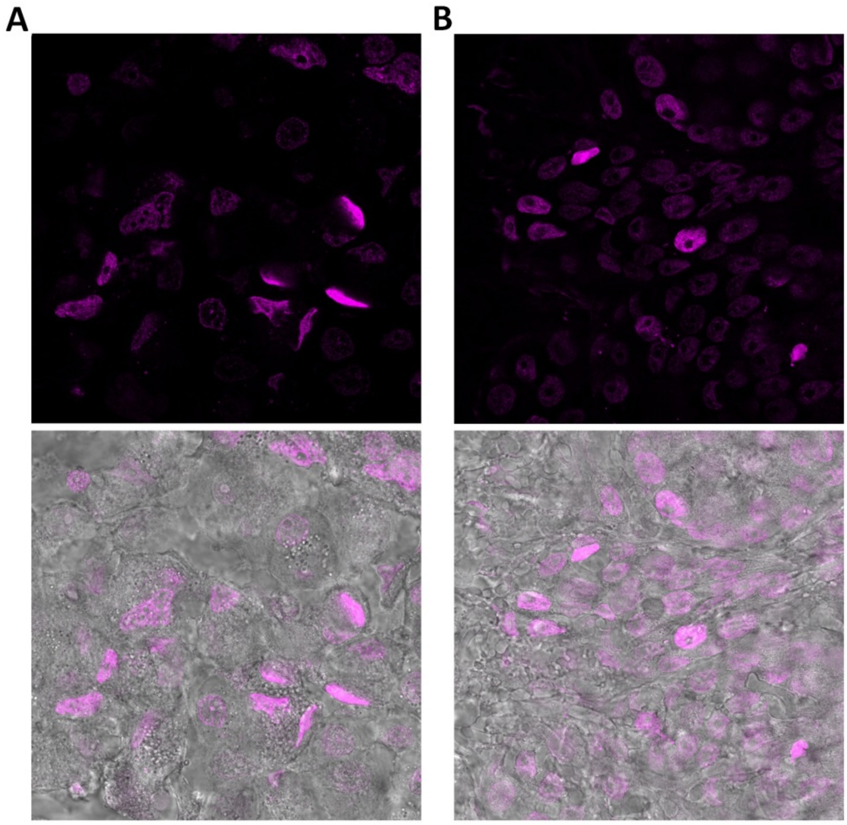

Supplementary Figure 7. Images of SKBR3 spheroids (Panel A) incubated with 2 µM doxorubicin for 48 hours and images of MCF10A spheroids (Panel B) incubated with 2 µM doxorubicin for 48 hours. The images below include the bright field images. Images were acquired at 100x magnification.

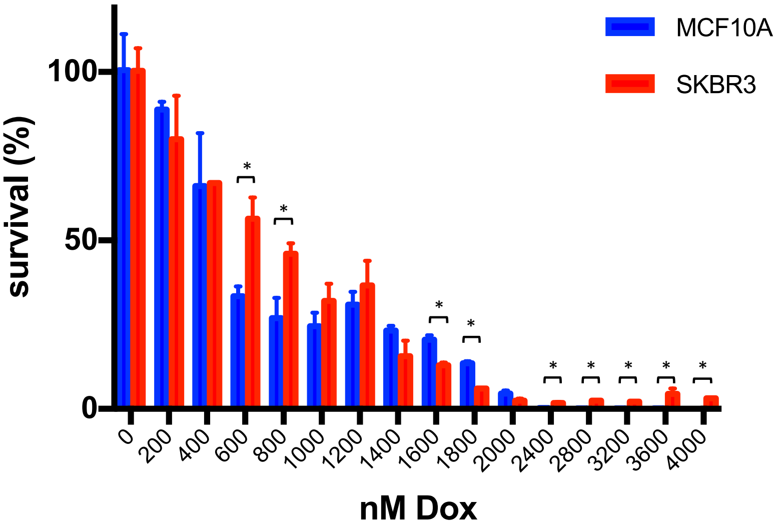

Supplementary Figure 8. Doxorubicin titration on cell viability of SKBR3 and MCF10A spheroids. Cell viabilities after 8 days of incubation with doxorubicin were calculated relative to untreated spheroids (0 µM doxorubicin). * P<0.01 MCF10A vs SKBR3 t-test. Discovery determined using the Two-stage linear step-up procedure of Benjamini, Krieger and Yekutieli, with Q = 1%.

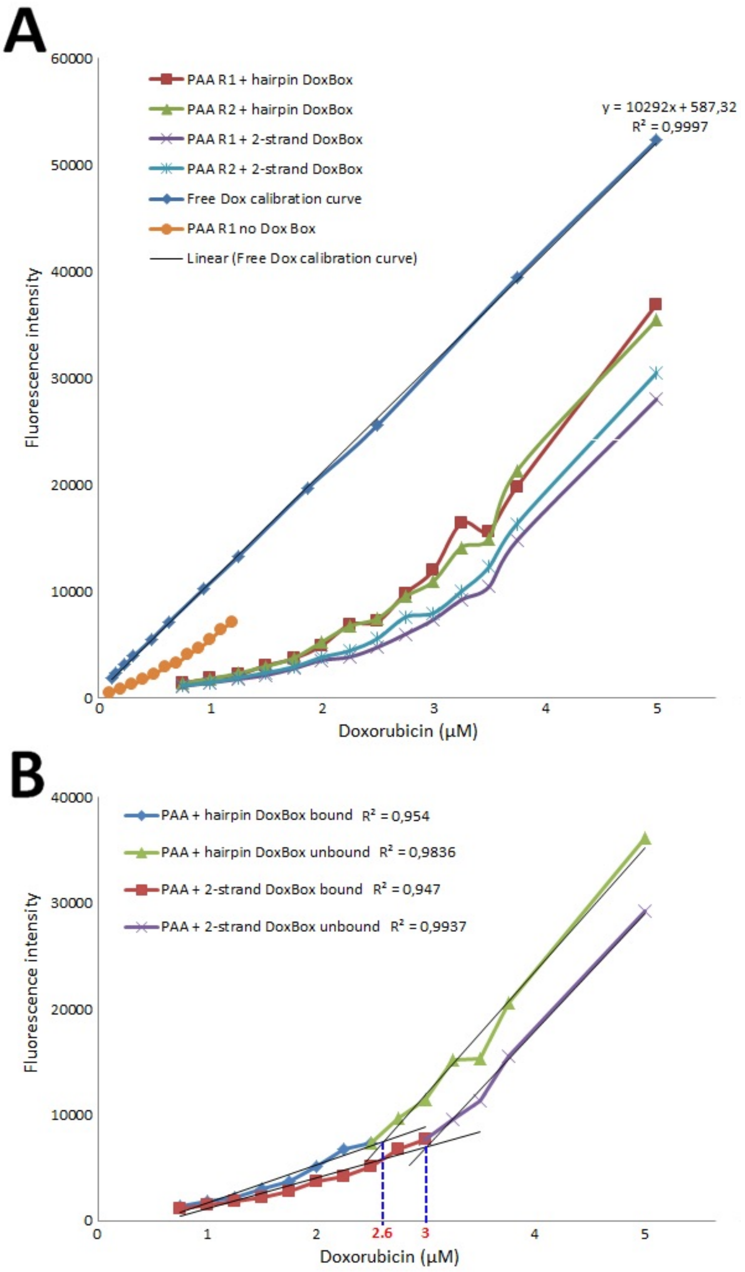

Supplementary Figure 9. A) Titration curves for the determination of the maximum doxorubicin load of the polymeric SKBR3-R1/2Tr aptamers with hairpin Dox boxes and 2-strand Dox boxes and polymeric SKBR3-R1Tr aptamers without Dox boxes. B) Determination of the inflection point between doxorubicin bound to DNA and doxorubicin free in solution. Titration curves were averaged per Dox box type and split in two linear halves. The upper half inclines in parallel to the calibration curve of free doxorubicin and the lower half inclines less steep due to fluorescence quenching. The cross section of the two straight lines is the approximated maximum doxorubicin charging capacity for the polymeric DNA samples. As Dox intercalates into double stranded DNA containing a CpG/GpC sequence, two binding sites are expected for the aptamer sequences.

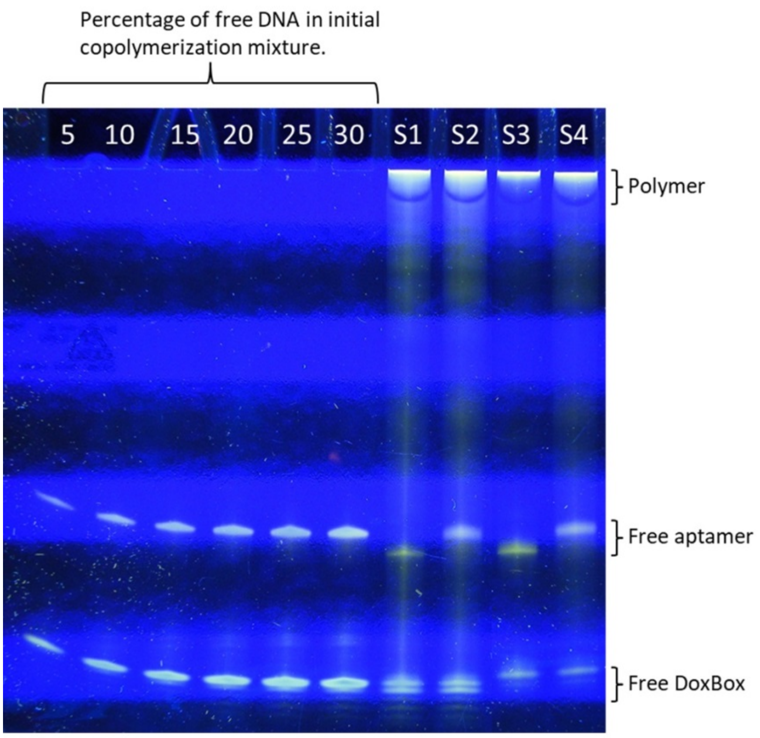

Supplementary Figure 10. Qualitative check on the degree of polymerisation using denaturing PAGE. Lanes S1 and S2 are respectively SKBR3-R1Tr and SKBR3-R2Tr co-polymerised with acrylamide and hairpin Dox boxes. Lanes S3 and S4 are respectively SKBR3-R1Tr and SKBR3-R2Tr co-polymerised with acrylamide and 2-strand DoxBox lead. Lanes 5 – 30 contain the amounts of free aptamer DNA and 2-strand DoxBox lead DNA that resemble a 95% – 70% polymerisation efficiency, respectively. Bands were visualized using SYBR gold.
